# Real-World Clinical Utility of Targeted RNA Sequencing in Leukemia Diagnosis and Management

**DOI:** 10.3390/cancers16132467

**Published:** 2024-07-05

**Authors:** Seo Wan Kim, Namsoo Kim, Yu Jeong Choi, Seung-Tae Lee, Jong Rak Choi, Saeam Shin

**Affiliations:** 1 Department of Laboratory Medicine, Yonsei University College of Medicine, Seoul 03722, Republic of Koreacjr0606@yuhs.ac (J.R.C.); 2 Dxome Co., Ltd., Seongnam-si 13558, Republic of Korea

**Keywords:** acute leukemia, RNA sequencing, karyotyping, gene fusion

## Abstract

**Simple Summary:**

This study explores the detection of gene fusions in acute leukemia using targeted RNA sequencing and compares its effectiveness with traditional methods like karyotyping and RT-PCR. Gene fusions, which result from chromosomal rearrangements, are crucial for diagnosing and treating leukemia. Recent updates in diagnostic criteria emphasize the importance of identifying these gene fusions. Targeted RNA sequencing offers advantages such as rapid diagnosis, minimal training requirements, and cost-effectiveness, making it a practical method in clinical settings. Our findings suggest that targeted RNA sequencing can accurately identify gene fusions, potentially benefiting nearly half of leukemia patients and improving diagnostic precision. This method may significantly impact the research community by providing a reliable and efficient tool for leukemia diagnosis.

**Abstract:**

Gene fusions are key drivers in acute leukemia, impacting diagnosis and treatment decisions. We analyzed 264 leukemia patients using targeted RNA sequencing with conventional karyotyping and reverse transcription polymerase chain reaction (RT-PCR). Leukemic fusions were detected in 127 patients (48.1%). The new guidelines introduced additional diagnostic criteria, expanding the spectrum of gene fusions. We discovered three novel fusions (*RUNX1::DOPEY2*, *RUNX1::MACROD2*, and *ZCCHC7::LRP1B*). We analyzed recurrent breakpoints for the *KMT2A* and *NUP98* rearrangements. Targeted RNA sequencing showed consistent results with RT-PCR in all tested samples. However, when compared to conventional karyotyping, we observed an 83.3% concordance rate, with 29 cases found only in targeted RNA sequencing, 7 cases with discordant results, and 5 cases found only in conventional karyotyping. For the five cases where known leukemic gene rearrangements were suspected only in conventional karyotyping, we conducted additional messenger RNA sequencing in four cases and proved no pathogenic gene rearrangements. Targeted RNA sequencing proved advantageous for the rapid and accurate interpretation of gene rearrangements. The concurrent use of multiple methods was essential for a comprehensive evaluation. Comprehensive molecular analysis enhances our understanding of leukemia’s genetic basis, aiding diagnosis and classification. Advanced molecular techniques improve clinical decision-making, offering potential benefits.

## 1. Introduction

Gene fusions can emerge from chromosomal rearrangements such as translocations, inversions, and deletions [1]. They play a vital role in classifying acute leukemia, choosing suitable therapy, and forecasting prognosis [2]. The fifth edition of the World Health Organization (WHO) classification [3,4] and the 2022 International Consensus Classification (ICC) [5,6] have recently provided revisions to the categorization of myeloid/lymphoid neoplasms and acute leukemias. One of the remarkable updates is a trend of introducing new gene rearrangements or mutations as diagnostic criteria. For instance, the *NUP98* rearrangement, which was not part of the acute myeloid leukemia (AML) with recurrent genetic abnormalities category in the fourth edition of the WHO classification [7], was introduced into the fifth edition of the WHO classification [3]. In addition, new subtypes of B-lymphoblastic leukemia (B-ALL) were added to the 2022 ICC, including *DUX4*, *ZNF384*, *MEF2D*, *MYC*, *NUTM1*, and *HLF* rearrangements [5]. When these gene rearrangements or mutations are detected, there is a decrease in the threshold of the blast cell percentage, which is considered a diagnostic criterion in leukemia. This change implies that rather than relying solely on morphological criteria, it is increasingly important to identify gene rearrangements or mutations that play a role in the progression of leukemia [8].

Conventional practices for detecting gene fusions involve chromosomal analysis, referred to as karyotyping, as well as fluorescence in situ hybridization (FISH) and reverse transcription polymerase chain reaction (RT-PCR) [9,10]. These techniques have limitations, including the potential to overlook hidden translocations due to low resolution, restricted coverage provided by pre-designed probes or primers aimed at known abnormalities, and a lack of multiplex capability [11]. Anchored Multiplex PCR (AMP) targeted RNA sequencing involves the development of amplicon-based cDNA libraries and has been introduced as an alternative option in contemporary laboratory settings [12]. AMP offers the application of various gene-specific primers (GSPs) and universal primers complementary to the molecular barcoded adaptor [13]. This approach allows amplification of target sequences without prior knowledge of the partner gene sequence.

The authors reported the clinical utility of targeted RNA sequencing in a small group of patients at an institution [12,14]. Based on this successful implementation of clinical targeted RNA sequencing, we conducted this study to investigate the clinical utility of targeted RNA sequencing in a larger patient cohort over several years. Furthermore, we compiled and compared studies that conducted validation studies using the 199-gene panel utilized in our paper [15,16,17]. In this retrospective study, we aim to confirm the clinical usefulness of targeted RNA sequencing in detecting recurrent leukemic fusion in clinical practice.

## 2. Materials and Methods

### 2.1. Patients and Samples

Patients initially diagnosed with acute leukemia in our hospital between July 2019 and October 2022 were included. Clinical information including patient age, sex, leukemia type, and initial blast cell percentage in bone marrow aspirate was collected. This study was approved with a waiver of informed consent by the Institutional Review Board (IRB) of Severance Hospital, Seoul, Korea (4-2023-0936), on 8 September 2023. Patient consent was waived due to the retrospective nature of the study and because the analysis used anonymous clinical data.

### 2.2. Conventional Genetic Assays

We compared targeted RNA sequencing results with conventional genetic assays, including G-banding karyotyping and qualitative RT-PCR. Heparinized bone marrow aspirate was cultured, harvested, and analyzed following standard protocols for G-banding karyotyping. The karyotype was described according to the International System for Human Cytogenetic Nomenclature. For RT-PCR, total RNA was extracted from the patients’ bone marrow aspirate using a QIAamp RNA Blood Mini Kit (Qiagen, Hilden, Germany). cDNA was synthesized using a Transcriptor First Strand cDNA Synthesis Kit (Roche Diagnostics, Indianapolis, IN, USA). RT-PCR was performed using a HemaVision 28N kit (DNA Technology A/S, Aarhus, Denmark) following the manufacturer’s instructions.

### 2.3. Targeted RNA Sequencing

The cDNA library was prepared from total RNA by two rounds of low-cycle PCR with an Archer FusionPlex Pan-Heme kit (ArcherDX, Boulder, CO, USA) using GSPs covering 199 target genes (Supplementary Materials Text S1) and universal primers. The final products were sequenced on a NextSeq 550Dx instrument (Illumina, San Diego, CA, USA), and data were analyzed and interpreted by Archer Analysis Software (version 5.1, ArcherDX). Leukemic fusions were selected from the observed in-frame fusions that have been recurrently reported in leukemia, based on the guidelines from the WHO and ICC as well as literature reports. The read count and read percentage were collected for the detected gene fusions. For in-depth assessment of *KMT2A* and *NUP98* gene rearrangements with multiple partner genes, breakpoint information was also collected. For cases in which known leukemic gene rearrangements were suspected from G-banding karyotyping but were not detected by targeted RNA sequencing, we confirmed them using messenger RNA (mRNA) sequencing (TruSeq Stranded mRNA; Illumina, CA, USA).

### 2.4. Statistical Analysis

The statistical analysis utilized R statistics version 3.6.3. We calculated the agreement between targeted RNA sequencing and conventional karyotyping using weighted kappa statistics [18]. The degree of agreement was classified as follows: 0.81–1.0, almost perfect or perfect agreement; 0.61–0.80, substantial agreement; 0.41–0.60, moderate agreement; 0.21–0.40, fair agreement; and <0.20, slight agreement.

## 3. Results

### 3.1. Patient Demographics

A total of 264 patients were enrolled in this study (Table 1). Patients were categorized into AML, B-ALL, T-lymphoblastic leukemia (T-ALL), and mixed phenotype acute leukemia (MPAL) groups, comprising 188, 69, 4, and 3 patients, respectively. The average age was higher in the AML group, with a value of 52.3 years, while those for the B-ALL, T-ALL, and MPAL groups were 12.7, 15.0, and 6.7, respectively. Leukemic fusions were detected in 127 patients (48.1%), with the lowest incidence observed in the AML group (46.3%). The average percentages of blast cells in bone marrow aspiration samples from the AML, B-ALL, T-ALL, and MPAL groups were 55.7%, 77.7%, 71.3%, and 87.0%, respectively.

### 3.2. Gene Rearrangement Results

We further analyzed the results of the 122 cases with targeted RNA sequencing (Table 2). A total of 36 distinct types of gene fusions were identified, with *RUNX1::RUNX1T1* being the most prevalent (21 cases), followed by *PML::RARA* (18 cases) and *ETV6::RUNX1* (15 cases).

**Figure 1:**
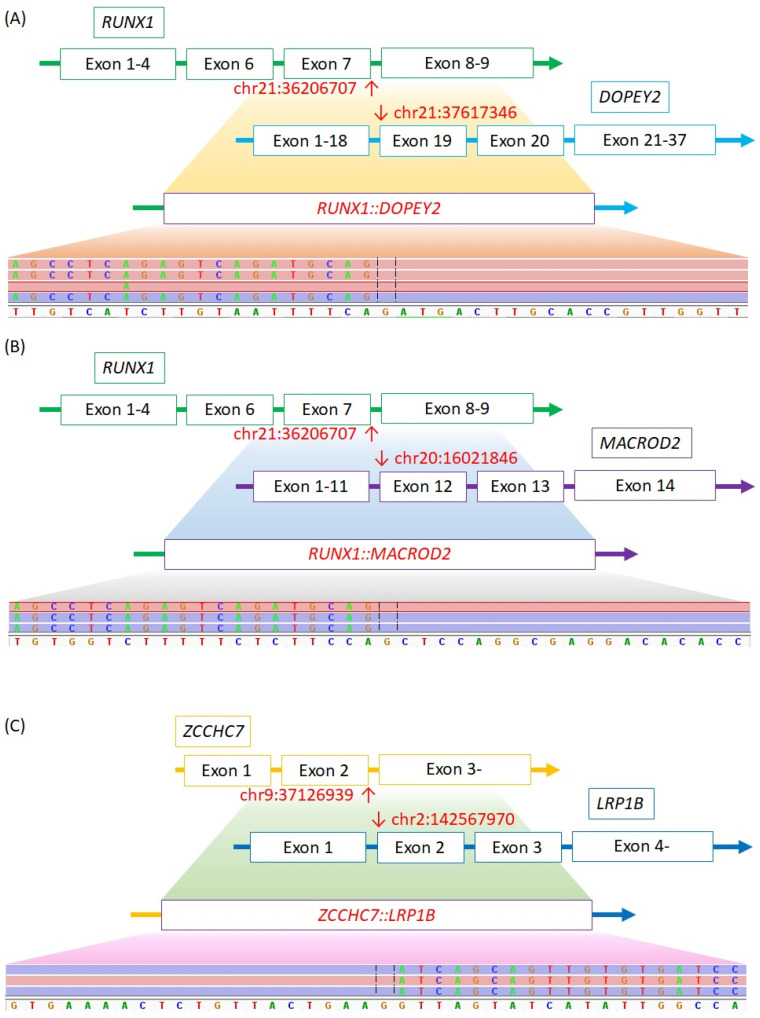
Schematic representation of the novel gene fusions: (**A**) *RUNX1::DOPEY2*, (**B**) *RUNX1::MACROD2*, (**C**) *ZCCHC7::LRP1B.*

In our study, three cases (*RUNX1::DOPEY2*, *RUNX1::MACROD2*, *ZCCHC7::LRP1B*) were speculated to be novel discoveries. All three fusions were analyzed to be in-frame (Figure 1A–C).

**Figure 2:**
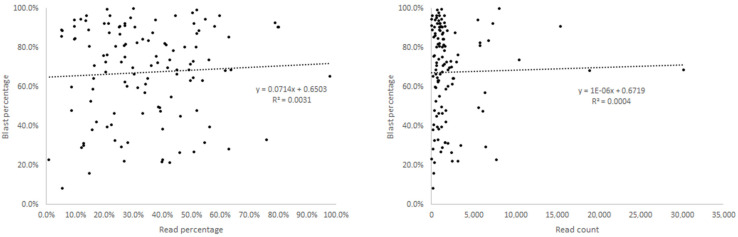
Comparison of the read percentage and read count in targeted RNA sequencing with the blast percentage.

Furthermore, we conducted an analysis of the read count and read percentage for detected gene fusions (Figure 2). There was no significant linear correlation observed between the total read percentage and the bone marrow aspiration blast percentage or between the total read count and the bone marrow aspiration blast percentage. Similarly, for the most frequent gene rearrangements of *BCR::ABL1*, *CBFB::MYH11*, *ETV6::RUNX1*, *PML::RARA*, and *RUNX1::RUNX1T1*, no significant linear correlation was found in their analysis (Figure S1).

**Figure 3:**
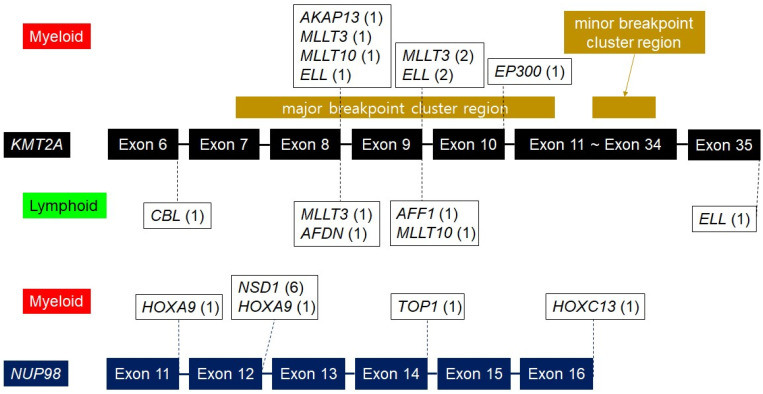
Partner genes and distribution of breakpoints in rearranged *KMT2A* and *NUP98* cases.

We analyzed how breakpoints are distributed in *KMT2A* and *NUP98*, which form fusions with many different partner genes (Figure 3). For *KMT2A*, breakpoints were most frequently observed between exon 8 and exon 10. All breakpoints for *NUP98* were situated between exon 11 and exon 16.

### 3.3. Comparison with Other Tests

We compared targeted RNA sequencing with the RT-PCR test (Table 3). A total of 62 RT-PCR tests were performed for *BCR::ABL1*, of which all (negative 55, positive 7) yielded consistent results with the targeted RNA sequencing. The 62 patients compared included 5 with AML, 52 with B-ALL, 2 with MPAL, and 3 with T-ALL. For *PML::RARA*, 43 tests were conducted, and all 26 negative and 17 positive results aligned with the targeted RNA sequencing.

Furthermore, we compared targeted RNA sequencing with conventional karyotyping (Table 4). A total of 246 cases had matchable results, of which 205 (83.3%) exhibited concordant findings with 78 positive and 127 negative matches for a known abnormality. Among these, there were 29 cases where conventional karyotyping indicated no known abnormality, but targeted RNA sequencing yielded positive results. Additionally, 12 cases showed a known abnormality in conventional karyotyping, but the targeted RNA sequencing did not provide accurate results. In 12 cases with inaccurate results, 9 cases showed complex chromosomal abnormalities.

There were five cases in which known abnormalities were suspected in conventional karyotyping but were not detected in targeted RNA sequencing. In the first case, t(1;19)(q21;p13.3), suspected as *TCF3::PBX 1* [19], we successfully detected this translocation in three patients but experienced failure in this specific case. The second case, t(7;21)(q22;q22), suspected as *RUNX1::USP42* [20], was detected in one patient with no detection in this specific case. In the first two cases, we observed that while we correctly detected fusions related to translocations in other patients, the cases where detection failed likely involved the same translocations as seen in karyotyping but involved different genes. In the third case, t(X;20)(q13;q13.3) was reported to be associated with leukemia at the chromosomal level. However, there have been no reports on the related gene [21]. As there have been no reports on the genes related to this translocation, it is presumed that they might not be present in the 199-gene panel. For the fourth case, t(12;12)(p12;p13), suspected as *TEAD4::C2CD5* [22], genes were not included in the 199-gene panel. Finally, the fifth case, t(5;14)(q31;q32), was estimated as *IGH::IL 3* [23]. There is a caveat to consider when performing targeted RNA sequencing using the Archer FusionPlex Pan-Heme kit. Fusions involving B cell receptor and T cell receptor loci, including *IGH*, *IGL*, and *IGK*, are targeted for expression and may not be explicitly called fusion because these often do not result in chimeric transcripts.

## 4. Discussion

In this study, we conducted a comprehensive analysis of gene fusions in different types of leukemia, evaluating their prevalence, distribution, and correlation with other diagnostic methods. Our results illuminate the intricate terrain of gene rearrangements and their significance in the context of leukemia categorization and identification.

Numerous studies have previously described the results of RNA sequencing in leukemia patients using their RNA [17]. In contrast, our study introduces several novel aspects. First, we compared targeted RNA sequencing with other examinations such as conventional karyotyping and RT-PCR. Second, we compared targeted RNA sequencing with the blast ratio in the patient’s bone marrow aspiration. Third, the frequency of gene rearrangement for each gene is clinically significant. We provided a comprehensive examination count, allowing us to determine the frequency of gene rearrangements for each specific gene.

Several studies, excluding our institution, have shown results using the Archer FusionPlex Pan-Heme kit we employed. To validate our findings, we compared and analyzed these studies. Chen et al. detected leukemic fusions in 37 out of 61 cases (60.7%) in their validation cohort and in 21 out of 28 cases (75%) in their unknown cohort suspected of having fusions [15]. Xu et al. detected leukemic fusions in 28 out of 87 AML cases (32.2%) [16]. Engvall et al. tested 25 out of 27 hematologic malignancy cases where chromosomal analysis and FISH had confirmed fusions, detecting leukemic fusions in all 25 cases [17]. While these studies were appropriate for validation cohorts, they often only included patients likely to have leukemic fusions, involved a small number of cases (under 100), or provided limited further evaluation of leukemic fusions. In contrast, our study includes all patients diagnosed with acute leukemia who underwent targeted RNA sequencing, ensuring a sufficient sample size. Additionally, we compared our results with other tests, analyzed blast percentage, and introduced several novel aspects through comprehensive analyses.

In total, among the 264 patients with leukemia, 122 (46.2%) were found to harbor gene fusions by targeted RNA sequencing, encompassing a total of 36 distinct types. When referencing previous findings, the prevalence of gene fusions in leukemia patients varied across panels, ranging from 30.0% to 60.0% [24,25]. Our research results are akin to these observations. In essence, this implies that approximately half of the patients possess gene fusions as driver mutations. Ongoing efforts involve utilizing gene fusions through targeted RT-PCR or targeted RNA sequencing to detect measurable residual disease (MRD) [26,27]. Based on our findings, it is anticipated that around half of the patients could derive benefits from assessing the presence of MRD through gene fusions.

We calculated the blast ratio from patient bone marrow aspirate samples and compared it with the read count and read percentage, illustrating the findings graphically. However, we observed no significant correlation between the blast ratio and read count or read percentage, which are fundamentally derived from RNA sequencing, obtained from transcriptionally active sources [28]. Consequently, gene fusion transcripts obtained from RNA sequencing might differ from the bone marrow blast composition [29]. In other words, even with a low blast ratio, the read count and read percentage derived from RNA sequencing could be substantial. Conversely, a high blast ratio might not necessarily indicate a large proportion of gene fusion clones, leading to a lower read count and read percentage. Furthermore, we conducted detailed analyses on frequently occurring gene fusions (*BCR::ABL1*, *CBFB::MYH11*, *ETV6::RUNX1*, *PML::RARA*, and *RUNX1::RUNX1T1*), but the results indicated a lack of relevance. We attempted to perform a similar analysis for RT-PCR; however, the hospital conducts qualitative rather than quantitative RT-PCR for newly diagnosed patients, which posed a limitation.

The WHO fifth edition introduced new diagnostic classifications, including “AML with *KMT2A* rearrangement” and “AML with *NUP98* rearrangement” [3]. We conducted additional analyses on the breakpoints of the *KMT2A* gene, revealing that the majority of breakpoints were located within the major breakpoint cluster (exon 7 to exon 13) [30]. Interestingly, one case of *KMT2A::CBL1* and one case of *KMT2A::ELL* had breakpoints that did not align with either major or minor clusters. According to previous research findings, the breakpoint of the *NUP98* gene was reported to be located from exon 8 to exon 14 [31,32]. Notably, while the *NUP98* gene comprises 33 exons, our findings indicate breakpoints between exon 11 and exon 16. Given that previous research has often reported breakpoints within this specific region, our results suggest its significance as a breakage hotspot within the *NUP98* gene.

RT-PCR, targeted RNA sequencing, and conventional karyotyping share several common aspects as diagnostic methods. However, considering the perspective of turnaround time (TAT), RT-PCR offers the fastest results, followed by targeted RNA sequencing and conventional karyotyping [33]. While the TAT for targeted RNA sequencing was initially 21 days in our institution, most results were obtained within approximately 14 days, indicating a favorable high-speed potential. Utilizing targeted RNA sequencing in our study allowed for superior outcomes compared to tests targeting fewer genes. Additionally, targeted RNA sequencing stands out by providing more detailed results compared to RT-PCR, which targets specific regions only, highlighting its advantages for precise AML diagnosis. Conventional karyotyping occasionally exhibits low resolution and might not reveal cryptic translocations, relying on 20 to 40 cultured metaphases for analysis. In this context, targeted RNA sequencing could greatly aid rapid and accurate interpretation of gene rearrangements. Our study results indicated high concordance among these three tests. However, discrepancies were also observed between them.

In our study, RT-PCR was performed for *BCR::ABL1* in 62 cases and for *PML::RARA* in 43 cases, with all cases showing concordance with targeted RNA sequencing. For these two fusions, rapid diagnosis is crucial, as it can lead to prescribing appropriate drugs and thereby improve patient prognosis [34,35]. Therefore, alongside targeted RNA sequencing, RT-PCR examination was necessary. Thus, in cases of B-ALL where *BCR::ABL1* testing can enable suitable drug therapy and minimal residual disease measurement, the concurrent use of *BCR::ABL1* RT-PCR is deemed essential.

We introduced Cohen’s kappa coefficient to accurately assess the concordance between conventional karyotyping and targeted RNA sequencing [18]. In conclusion, there was substantial agreement between the total patient group and AML but only fair agreement in B-ALL. Cryptic chromosomal aberrations are common in ALL, and various methods, such as fluorescence in situ hybridization, have been proposed to detect them [36].

Among the 12 cases with inaccurate results, 9 cases had complex chromosomal abnormalities, which can interfere with RNA sequencing. In 5 of these 12 cases, gene fusions could not be detected at all. These cases suggest the potential insufficiency of our panel genes. Therefore, it is suggested that more comprehensive approaches, such as total RNA sequencing, whole genome sequencing, or optical mapping [37], which are capable of covering a broader range of genes, may be required to address cases that were not identified by either conventional karyotyping or gene fusion analysis alone. Meanwhile, whole genome sequencing and optical genome mapping are emerging as novel technologies compared to current targeted RNA sequencing, with limitations such as high cost and current application mainly limited to germline mutations. In contrast, targeted RNA sequencing offers advantages of low server capacity, rapid readout speed, and cost-effectiveness.

In our case, to address this issue, we proceeded with additional mRNA sequencing. Regarding adding some genes to the existing panel for additional analysis, we concluded that the current panel of 199 genes holds sufficient data for frequently occurring genes, and to cover even less common genes, a switch to whole RNA sequencing would be necessary. As evident from the results of the four cases, while recurrent abnormal karyotypes were detected at the chromosomal level, it was confirmed through mRNA sequencing that there were variants of unknown significance, namely fusion or no mRNA-based fusion. Two key outcomes emerged: firstly, the findings that pathogenic fusions were not discovered in the four cases by targeted RNA sequencing were accurate; and secondly, the importance of RNA-based methods in leukemia was emphasized to complement chromosomal examinations that are challenging to confirm at the gene level. However, the mRNA sequencing we conducted required more than 5–10 times the server capacity compared to targeted RNA sequencing, had a longer processing time, and was associated with higher costs.

Based on practical experience in the laboratory with both targeted RNA sequencing and mRNA sequencing, several advantages unique to targeted RNA sequencing were observed. Firstly, fusions that are not detectable by karyotyping can be rapidly diagnosed, allowing patients to receive an accurate diagnosis according to updated diagnostic criteria. Additionally, as mentioned earlier, the server capacity for targeted RNA sequencing is sufficient to handle a large number of patients without causing delays or interference with other tests. Secondly, targeted RNA sequencing can be quickly adopted in clinical practice because it requires only minimal training for both the operators and interpreters to conduct accurate tests. Economically, targeted RNA sequencing also holds an advantage.

It is important to acknowledge the limitations of this study. First, the patient cohort was exclusively obtained within a single medical center, potentially introducing bias and limiting the generalizability of the findings to a broader population. Notably, since this was a retrospective study conducted at a single center, the number of T-ALL and MPAL cases was small, which could introduce a bias in case proportions. Additionally, the retrospective nature of the study design may have inherent limitations in data collection and potential for uncontrolled confounding variables. While the results provide valuable insights into the context of gene fusion analysis and its implications, these constraints underscore the need for further multicenter prospective studies to corroborate and extend the present findings to a more diverse patient population.

## 5. Conclusions

In conclusion, we identified recurrent leukemic fusion genes in 48.1% of patients using targeted RNA sequencing. Rearrangements involving genes of *KMT2A* and *NUP98* which have multiple fusion partners were also successfully identified from targeted RNA sequencing. RT-PCR, NGS, and karyotyping showed distinct diagnostic strengths, while comprehensive approaches were considered. Given the necessity of testing for leukemic fusions in the diagnosis of leukemia, rapid and accurate targeted RNA sequencing, despite its limitations of rare discordance with conventional methods and absence of correlation with blast counts, could be a highly effective method in the laboratory setting.

## Figures and Tables

**Table 1:** Demographic features of patients (*n* (%) or average (SD)).

	AML (*n* = 188)	B-ALL (*n* = 69)	T-ALL (*n* = 4)	MPAL (*n* = 3)
Male sex	96 (51.1)	36 (52.2)	3 (75.0%)	1 (33.3)
Age	52.3 (22.4)	12.7 (16.5)	15.0 (10.6)	6.7 (4.6)
Leukemic fusion	87 (46.3)	36 (52.2)	2 (50.0)	2 (66.7)
Percentage of blasts	55.7 (24.5)	77.7 (22.3)	71.3 (34.8)	87.0 (20.0)

Abbreviations: AML: acute myeloid leukemia; B-ALL: B-lymphoblastic leukemia; MPAL: mixed phenotype acute leukemia; T-ALL: T-lymphoblastic leukemia.

**Table 2:** Gene fusions detected in targeted RNA sequencing.

	AML	B-ALL	T-ALL	MPAL	Total
*BCR::ABL1*	2	6			8
*BCR::ABL1*, *PAX5::ZCCHC7*		1			1
CBFA2T3::GLIS2 *	1				1
*CBFB::MYH11*	10				10
*DEK::NUP214*	3				3
*EBF1::PDGFRB*		1			1
EP300::ZNF384 *		1			1
*ETV6::RUNX1*		15		1	16
*FNDC3B::MECOM*	1				1
FUS::ERG *	1				1
*KMT2A::AFDN*		1			1
*KMT2A::AFF1*		1			1
KMT2A::AKAP13 *	1				1
*KMT2A::CBL*		1			1
KMT2A::EL L *	3		1		4
KMT2A::EP300 *	1				1
KMT2A::MLLT10 *	1	1			2
KMT2A::MLLT3 *	3	1			4
*NUP214::ABL1*			1		1
NUP98::HOXA9 *	2				2
NUP98::HOXC13 *	1				1
NUP98::NSD1 *	6				6
NUP98::TOP1 *	1				1
*P2RY8::CRLF2*	1	1			2
*PAX5::C20orf112*, *P2RY8::CRLF2*		1			1
PICALM::MLLT10 *	1				1
*PML::RARA*	18				18
*RUNX1::DOPEY2*	1				1
*RUNX1::MACROD2*	1				1
*RUNX1::MECOM*	1				1
*RUNX1::RUNX1T1*	20			1	21
*RUNX1::USP42*	1				1
*SET::NUP214*	1				1
*TCF3::HLF*		1			1
*TCF3::PBX1*		3			3
*ZCCHC7::LRP1B*		1			1
Total	82	36	2	2	122

Abbreviations: AML: acute myeloid leukemia; B-ALL: B-lymphoblastic leukemia; MPAL: mixed phenotype acute leukemia; T-ALL: T-lymphoblastic leukemia. *: newly defined recurrent genetic abnormalities in the WHO fifth edition or ICC. *EP300::ZNF384* was B-lymphoblastic leukemia, while the others were all acute myeloid leukemia.

**Table 3:** Comparison of RT-PCR and targeted RNA sequencing.

		RT-PCR			
		*BCR::ABL1*		*PML::RARA*	
		Negative	Positive	Negative	Positive
RNA seq	Not detected	55	0	26	0
	Detected	0	7	0	17

**Table 4:** Comparison of conventional karyotyping and targeted RNA sequencing.

		Karyotyping			Cohen’s Kappa
RNA Seq		Known Abnormality	No Known Abnormality	Not Applicable	
Total	Matched	78	29	8	0.655
	Not matched	7	0	0	
	Not detected	5	127	10	
AML	Matched	64	13	2	0.760
	Not matched	3	0	0	
	Not detected	5	95	6	
B-ALL	Matched	12	14	6	0.355
	Not matched	4	0	0	
	Not detected	0	29	4	
T-ALL	Matched	1	1	0	0.500
	Not matched	0	0	0	
	Not detected	0	2	0	
MPAL	Matched	1	1	0	0.400
	Not matched	0	0	0	
	Not detected	0	1	0	

Abbreviations: AML: acute myeloid leukemia; B-ALL: B-lymphoblastic leukemia; MPAL: mixed phenotype acute leukemia; T-ALL: T-lymphoblastic leukemia.

## Data Availability

All data generated or analyzed during this study are included in this published article and its Supplementary Materials.

## Supplementary Materials

The following supporting information can be downloaded at https://www.mdpi.com/article/10.3390/cancers16132467/s1, Text S1. The list of 199 target genes of the Archer FusionPlex Pan-Heme kit (ArcherDX, Boulder, Colorado), Figure S1. Comparison of the read percentage in NGS fusion with the blast percentage within (A) *BCR::ABL1*, (B) *CBFB::MYH11*, (C) *ETV6::RUNX1*, (D) *PML::RARA*, and (E) *RUNX1::RUNX1T1*. Comparison of the read count in NGS fusion with the blast percentage within (F) *BCR::ABL1*, (G) *CBFB::MYH11*, (H) *ETV6::RUNX1*, (I) *PML::RARA*, and (J) *RUNX1::RUNX1T1*.
