# Supplementary material for: Real-World Clinical Utility of Targeted RNA Sequencing in Leukemia Diagnosis and Management
Source: Cancers (Basel). 2024 Jul 5;16(13):2467. doi: 10.3390/cancers16132467 (PMC11240350; doi:10.3390/cancers16132467)
Supplement: Supplementary file 1 [file cancers-16-02467-s001.zip › cancers-3056722-supplementary.pdf]

Supplementary Materials: Real-World Clinical Utility of Targeted RNA Sequencing in Leukemia Diagnosis and Management

Seo Wan Kim, Namsoo Kim, Yu Jeong Choi, Seung-Tae Lee, Jong Rak Choi and Saeam Shin

Text S1. The list of 199 target genes of the Archer FusionPlex Pan-Heme kit (ArcherDX, Boulder, Colorado).

ABL1, ABL2, AICDA, AKT3, ALK, ASB13, ASXL1, BATF3, BAX, BCL11B, BCL2, BCL2A1, BCL3, BCL6, BCR, BIRC3, BLNK, BMF, BMP7, BRAF, BTK, CALR, CARD11, CBFB, CBL, CCDC50, CCND1, CCND2, CCND3, CD274, CD44, CD79B, CDC25A, CDK6, CDKN2A, CDKN2B, CEBPA, CEBPD, CEBPE, CEBPG, CHD1, CHIC2, CIITA, CREB3L2, CREBBP, CRLF2, CSF1R, CSF3R, CTLA4, CYB5R2, DCK, DEK, DENND3, DLEU1, DNM2, DNMT3A, DNMT3B, DNTT, DUSP22, E2F2, EBF1, EIF4A1, ENTPD1, EPOR, ERG, ETV6, EXOC2, EZH2, FAM216A, FBXW7, FGFR1, FGFR2, FGFR3, FLT3, FOXP1, FUT8, GATA1, GATA2, GLIS2, GNAS, HOXA10, HOXA9, ID4, IDH1, IDH2, IKZF1, IKZF2, IKZF3, IL16, IL7R, IRF4, IRF8, ITPKB, JAK1, JAK2, JAK3, KAT6A, KDM6A, KIAA0101, KIT, KLF2, KMT2A, KRAS, LIMD1, LMO1, LMO2, LRMP, LYL1, LZTS1, MAL, MALT1, MAML3, MECOM, MKL1, MLF1, MLLT10, MLLT4, MME, MPL, MUC1, MYBL1, MYC, MYD88, MYH11, NEK6, NF1, NFKB1, NFKB2, NME1, NOTCH1, NOTCH2, NPM1, NRAS, NT5C2, NTRK3, NUP214, NUP98, P2RY8, PAG1, PAICS, PAX5, PBX1, PDCD1, PDCD1LG2, PDGFRA, PDGFRB, PHF6, PICALM, PIM1, PIM2, PLCG1, PLCG2, PML, PPAT, PRDM16, PRKAR2B, PTK2B, PTPN1, PTPN11, PYCR1, RAB7L1, RAG1, RAG2, RANBP1, RARA, RBM15, RHOA, ROS1, RUNX1, RUNX1T1, S1PR2, SEMA6A, SERPINA9, SETBP1, SETD2, SF3B1, SH2B3, SH3BP5, SLC29A1, SOX11, SRSF2, STAT3, STAT5B, STAT6, STIL, STRBP, TAL1, TCF3, TFG, TLX1, TLX3, TNFRSF13B, TNFSF4, TP63, TYK2, U2AF1, WT1, XPO1, ZCCHC7

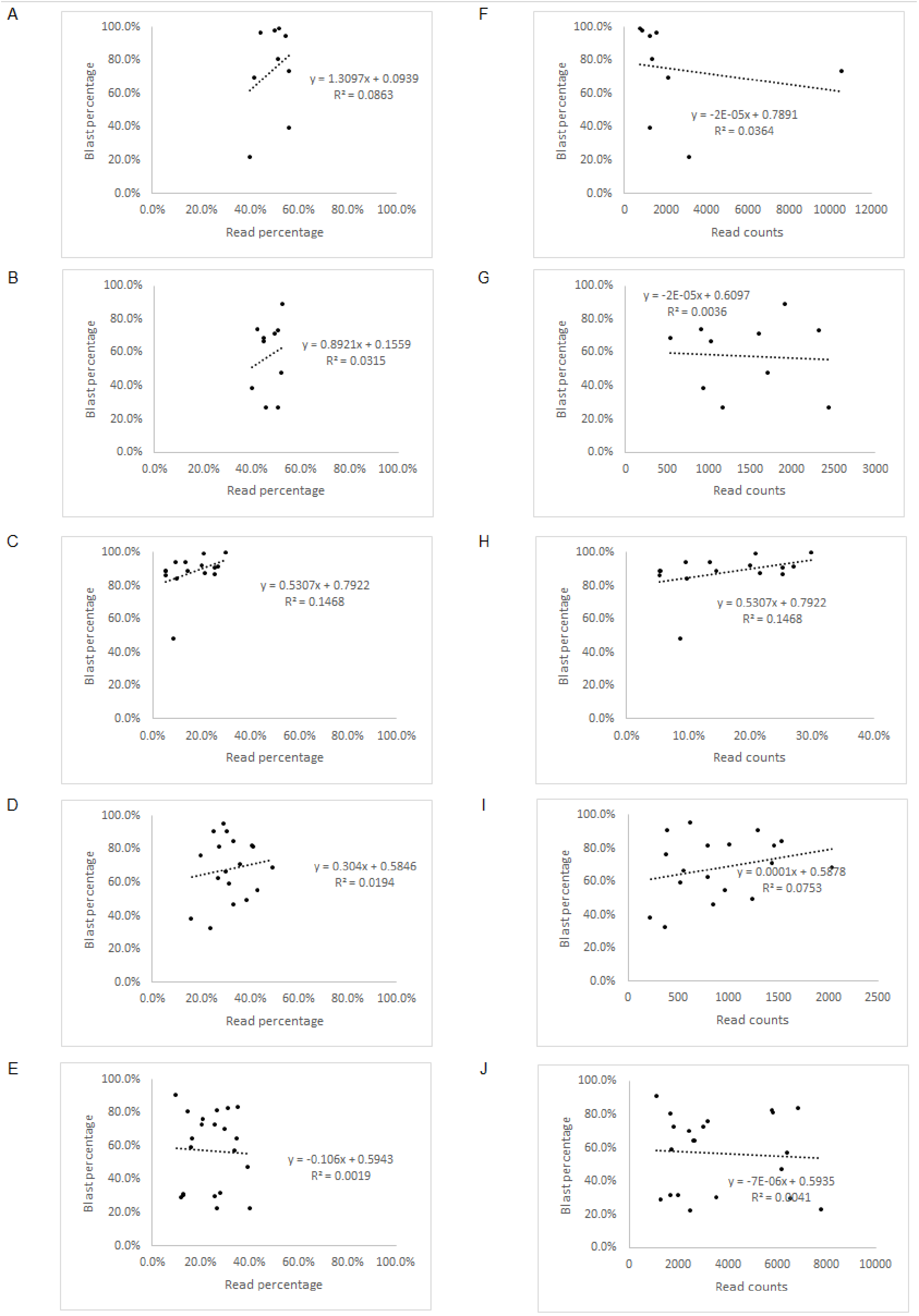
A
Blast percentage
Read percentage
y = 1.3097x + 0.0939
R² = 0.0863
B
Blast percentage
Read percentage
y = 0.8921x + 0.1559
R² = 0.0315
C
Blast percentage
Read percentage
y = 0.5307x + 0.7922
R² = 0.1468
D
Blast percentage
Read percentage
y = 0.304x + 0.5846
R² = 0.0194
E
Blast percentage
Read percentage
y = -0.106x + 0.5943
R² = 0.0019
F
Blast percentage
Read counts
y = -2E-05x + 0.7891
R² = 0.0364
G
Blast percentage
Read counts
y = -2E-05x + 0.6097
R² = 0.0036
H
Blast percentage
Read counts
y = 0.5307x + 0.7922
R² = 0.1468
I
Blast percentage
Read counts
y = 0.0001x + 0.5878
R² = 0.0753
J
Blast percentage
Read counts
y = -7E-06x + 0.5935
R² = 0.0041

Figure S1. Comparison of the read percentage in NGS fusion with the blast percentage within (A) *BCR::ABL1*, (B) *CBFB::MYH11*, (C) *ETV6::RUNX1*, (D) *PML::RARA*, and (E) *RUNX1::RUNX1T1*. Comparison of the read count in NGS fusion with the blast percentage within (F) *BCR::ABL1*, (G) *CBFB::MYH11*, (H) *ETV6::RUNX1*, (I) *PML::RARA*, and (J) *RUNX1::RUNX1T1*.
